# The Role of Neoantigens in Cancer Immunotherapy

**DOI:** 10.3389/fonc.2021.682325

**Published:** 2021-08-27

**Authors:** Yueting Zhu, Jiyan Liu

**Affiliations:** ^1^Department of Biotherapy, Cancer Center, and National Clinical Research Center for Geriatrics, West China Hospital, West China Medical School, Sichuan University, Chengdu, China; ^2^Department of Oncology, The First People’s Hospital of Ziyang, Ziyang, China

**Keywords:** neoantigen, immunotherapy, vaccine, adoptive cell therapy, personalized treatment

## Abstract

Somatic mutation-derived neoantigens, expressed only on tumor cells, may elicit antitumor T-cell responses in cancer immunotherapies with minimal immune tolerance. Neoantigens can be identified by multiple bioinformatics technologies, mainly based on whole-exome sequencing. Personalized cancer vaccines and adoptive T cell therapies are two primary treatment modalities targeting neoantigens, and both of them have shown promising therapeutic effects. This review, summarizes the history of neoantigen-related tumor control, introduces recent neoantigen screening and identification methods, and discusses the role of neoantigen in cancer immunotherapies. Moreover, we propose the challenges of targeting neoantigens for cancer treatment.

## Introduction

Immunotherapy has revolutionized the management of cancer treatment. Targeting the immune inhibitory regulators PD-1, PD-L1 or CTLA-4 is a common way to promote antitumor immune responses. However, immunotherapy boosting the immune system to destroy cancer cells has shown durable clinical responses in patients with various malignancies, but only in a subset of patients (1). Moreover, the non-antigen-specific simulation can activate global T cells and damage the human body, leading to immune-related adverse events, even fatal events (2). Thus substantial efforts are needed to explore more specific and powerful immunotherapy approaches, either alone or in combination.

As a genetic disease, cancer results from the accumulation of DNA damage and genetic alterations (3). These non-synonymous somatic mutations generate neoepitopes, which can be recognized by endogenous T cells as non-self-proteins and induce an antitumor immune response (1, 4). In most cases, neoantigens arise from single nucleotide mutations (SNV), gene fusion, alternative splicing, intron retention, insertion, and deletions. Other sources of neoantigens including post-translational modifications and endogenous retrovirus-associated tumors (5). The presentation of neoantigens is tumor-specific and able to elicit T cell-mediated antitumor immunity (4). Therefore, neoantigen is an ideal immunotherapy candidate. Two primary neoantigen-based therapeutic modalities have been explored in clinical practices: personalized vaccines and adoptive cell therapy (ACT) (6). Here we review the history of neoantigens about tumor control and introduce recent neoantigen screening and identification methods. We also discuss the role of neoantigen in cancer immunotherapies and its current challenges.

## Historical Overview of Tumor Neoantigens

The foundation of cancer immunology can date back to the middle of the twentieth. Gross (7) reported that transplantable tumors could produce active immunity against syngeneic mice. Another research showed that carcinogen-induced tumors had antigenic properties that the immune system could identify (8). In 1988, De Plaen et al. (9) discovered the first neoantigen recognized by cytolytic T lymphocytes (CTLs) in a methylcholanthrene (MCA)-induced mouse tumor model. The recognized neoantigen also could be identified in a cDNA library. Then, CD4+ T cells stimulated tumor-specific immunity and inhibited tumor growth after recognizing neoantigens in mice (10). In 1996, mutated neoantigens recognized by tumor-infiltrating lymphocytes (TILs) and CTLs were found in human melanoma (11) and renal cell carcinoma (12), respectively. Several years later, Rosenberg and his colleagues (13) revealed nearly complete regression in a melanoma patient after receiving autologous tumor-reactive TILs. T cells reactivity against neoantigen was proved to predominate cellular antitumor response in a long-term surviving melanoma patient (14). A detailed analysis in a melanoma patient with complete tumor regression after adoptive TILs transfer indicated that tumor-reactive T cells could be persistent and relevant for tumor regression (15). Collectively, neoantigen has the potential to be the target of antitumor immunity.

Advances in next-generation sequencing (NGS) technologies have provided access to compare mutations in the normal and tumor genome in 2008 (16). Then NGS was used to demonstrate tumor-specific antigens in an immunogenic mouse model, which opened a new dimension for antigenic targets of cancer immunity (17). By using NGS, Castle et al. (18) assessed the antitumoral activity of the neoantigen vaccine in the B16 melanoma tumor model. Consequently, the vaccine-elicited T-cell immunogenicity showed protective effects, providing new insights into immunogenic neoantigen-based vaccine treatment in human cancers (18). NGS also contributed to identify neoantigens recognized by TILs in melanoma patients (19, 20) and suggested the feasibility of neoantigen-specific T cell reactivity analysis in human cancer immunotherapy (20). Neoantigen was identified as the target of checkpoint blockade immunotherapies in a sarcomas mouse model (21). Concurrently, the adoptive transfer of neoantigen-specific CD4+ T cells achieved tumor regression in a metastatic epithelial cancer (22).

Further investigations found that neoantigen burden was associated with immune checkpoint inhibitors’ therapeutic responses in melanoma (23) and lung cancer patients (24). In 2015, Beatriz’s team first reported that vaccination with neoantigen could augment T cell immunity in patients with advanced melanoma, demonstrating the efficacy and feasibility of a personalized dendritic cell (DC)vaccine (25). A subsequent study revealed that ACT targeting mutant KRAS neoantigen mediated antitumor immune system, leading to regression in patients with metastatic colon cancer (26). Furthermore, the correlation between clonal neoantigen burden and response to immune checkpoint blockade was defined, rendering it is possible to target clonal neoantigens in T cell therapies (27). Ott et al. treated melanoma patients with a peptide vaccine targeting 20 predicted personal tumor neoantigens (28). Consistent with the reactive T cells responses, four patients had no recurrent diseases, and two patients had complete tumor regression after anti-PD-1 was administrated to recurrence (28). Then individualized RNA mutanome vaccine also induced T cell infiltration and killed autologous tumor cells, resulting in tumor regression and durable survival (29). The personalized neoantigen vaccine and nivolumab combination proved safe and immunogenicity in advanced solid tumors (30).

## Identification of Tumor-Specific Neoantigens

With the advances of NGS, it has become feasible to acquire genomic changes in some kinds of tumors, which provided the basis of neoantigen identification (31). Thus, the current process of predicting candidate neoantigens mainly includes three parts: 1) identify tumor-specific mutations using whole-genome sequencing (WGS), whole-exome sequencing (WES), or RNA-seq; 2) predict major histocompatibility complex (MHC) types, which in humans are called human leukocyte antigen (HLA) and neoantigen presentation; 3) prioritize and select candidate neoantigens (4, 32).

WES should be performed to map the cancer mutanome from the tumor and paired normal tissue. Combining RNA-seq can determine whether the mutation is expressed in tumors and infer its relative frequency overlapped with exome-based variants (4, 33). The minimum requirements for immunogenic and potential therapeutic mutations include: 1) the mutant peptide must be processed and presented by MHC molecules, and 2) the presented peptide-MHC complex must be recognized by endogenous T cells (31). Antigen processing and presentation are complex but different processes for MHC class I and II molecules (34). Peptides binding to MHC-I molecules are usually of a small length of 8-10 residues, while MHC-II molecules could bind to longer peptides with a length of 11-20 amino acids (34–36). Several tools depend on NGS data from WES, WGS, or RNA-seq can be used to predict HLA alleles, including Optitype (37) and Polysolver (38) for class I alleles, seq2HLA (39), Athlates (40), HLAScan (41), HLAProfiler (42), PHLAT (43), and ArcasHLA (44) for both class I and II HLA alleles. Following this, computational algorithms [for example, NetMHC (45), NetMHCpan (46, 47), and MHCflurry (48)] have been developed to predict MHC binding affinity. The criteria of binding affinities (IC50) are defined as high, intermediate to weak, and non-binder with IC50 <150, 150-500, or >500 nM, respectively (the commonly described binding affinity threshold) (49). The prioritization of candidate neoantigens can be achieved by the predicted binding affinities alone or in combination with the mutant expression level (50). On the other hand, mass spectrometry (MS) based immunopeptidome has enabled the discovery of thousands of MHC-associated neoantigens (51), and the combination of MS and WES is a powerful weapon to predict immunogenic tumor mutations (52). Compared to MS, T cell-based assays can directly detect whether an MHC-presented neoantigen has been recognized by T cells (4, 32). A variety of methodologies [e.g., fluorescently labeled HLA tetramers or multimers (53), the enzyme-linked immunosorbent spot (ELISpot) (54)] that stimulate and test neoantigen-reactive T cells have been reported. However, these cell-based assays are currently costly, time-consuming, and technically challenging (4).

## Clinical Application of Neoantigens

The predicted tumor-specific neoantigens are relevant targets for clinical personalized immunotherapies, either as a vaccine or a cellular therapy product.

### Vaccines

Personalized vaccines can be formulated as synthetic long peptide (SLP), DNA, RNA, DC, and viral and bacteria (55). Results from several clinical trials using neoantigen vaccines in patients with melanoma or glioblastoma are encouraging (25, 28, 29, 56, 57). Carreno et al. (25) first found that the DC vaccine augmented pre-existing neoantigen-specific immunity and induced previously undetected neoantigen induced T cell responses in three advanced melanoma patients. Neoantigens are processed and presented by HLA-A*02:01 molecules. Two subsequent clinical studies published in 2017 confirmed the potential of personalized neoantigen vaccine in treating patients with melanoma (28, 29). Ott et al. vaccinated six patients with SLP targeting up to 20 predicted tumor neoantigens (NCT01970358). Four of the six patients experienced no tumor recurrence in the following 25 months after vaccination, and another two patients with recurrence achieved complete tumor regression after received anti-PD-1 therapy (28).

Further analysis indicated that eight high-risk patients had durable neoantigens-induced T cell responses. It is encouraging that almost four years after being treated with vaccines, all are alive, and six have no active disease (58). Sahin et al. generated the first individualized RNA mutanome vaccines in 13 patients with stage melanoma (NCT02035956). Eight patients remained tumor-free within the follow-up period. Two of five patients with metastatic disease achieved objective responses, while one presented a complete response to RNA vaccine combined with PD-1 inhibitor (29).

Notably, two recent clinical trials emphasized the significance of the tailored vaccines in treating glioblastoma (56, 57). First, Keskin et al. treated glioblastoma patients with an individualized multi-epitope vaccine (NCT02287428), leading to increased neoantigen-specific CD4+ and CD8+ T cells responses in peripheral blood (56). Second, in another clinical trial, Hilf et al. reported that the combination of personalized vaccine and standard of care (NCT02149225) could induce sustained CD8+ T cells responses and predominantly CD4+ Th1 cell responses in glioblastoma patients (57).

Those encouraging results indicated that the personalized neoantigen vaccine approach is feasible for immunologically “cold” tumors with a low tumor mutation burden (56). Another early DC-based vaccine study in ovarian cancer has shown promising clinical outcomes without serious adverse events (59). Vaccination upregulated T cells responses against neoantigens, therefore elicited a broad antitumor immunity. In line with previous studies, the neoantigen-based EpiGVAX vaccine improved antitumor immunity in colorectal cancer (60). Recently, Ott et al. conducted a clinical trial that combined personalized neoantigen-based vaccine (NEO-PV-01) with nivolumab in patients with advanced melanoma, non-small cell lung cancer, and urothelial cancer (NCT02897765) (30). T cells responsive to neoantigens were detected in all vaccinated patients, while no serious adverse events were observed. The increasing T cells induced by this approach could control tumor growth and kill tumor cells, leading to potential clinical benefits (30).

To date, dozens of clinical trials investigating personalized neoantigen-based vaccines alone or in combination with checkpoint inhibitors are underway in various cancers (**Table 1**).

**Table 1:** Clinical trials of neoantigen vaccines.

ClinicalTrial.gov identifier	Phases	Enrollment status	Cancer type	Vaccine format	Additional intervention	Patient accrual target
NCT03558945	Phase 1	Recruiting	Pancreatic tumor	peptide	None	60
NCT04487093	Phase 1	Recruiting	Non small cell lung cancer	peptide	EGFR-TKI/anti-angioge	20
NCT04397926	Phase 1	Recruiting	Non small cell lung cancer	peptide	None	20
NCT02950766	Phase 1	Recruiting	Renal cell carcinoma	peptide	Ipilimumab	19
NCT03359239	Phase 1	Recruiting	Urothelial cancer	peptide	Atezolizumab	15
NCT02287428	Phase 1	Recruiting	Glioblastoma	peptide	Pembrolizumab	56
NCT03956056	Phase 1	Recruiting	Pancreatic cancer	peptide	None	15
NCT04117087	Phase 1	Recruiting	Colorectal cancer, pancreatic cancer	peptide	Nivolumab, ipilimumab	30
NCT04248569	Phase 1	Recruiting	Hepatocellular carcinoma	peptide	Nivolumab, ipilimumab	12
NCT04072900	Phase 1	Recruiting	Melanoma	peptide	Toripalimab	30
NCT03953235	Phase 1/2	Recruiting	Solid tumors	peptide	Nivolumab, ipilimumab	144
NCT03639714	Phase 1/2	Recruiting	Solid tumors	peptide	Nivolumab, ipilimumab	214
NCT04161755	Phase 1	Recruiting	Pancreatic cancer	peptide	Atezolizumab, mFOLFIRINOX	20
NCT04024878	Phase 1	Recruiting	Ovarian cancer	peptide	Nivolumab	30
NCT03552718	Phase 1	Recruiting	Solid tumors	peptide	None	16
NCT04251117	Phase 1/2	Recruiting	Hepatocellular carcinoma	DNA	Pembrolizumab	24
NCT03199040	Phase 1	Recruiting	Triple negative breast cancer	DNA	Durvalumab	24
NCT04015700	Phase 1	Recruiting	Glioblastoma	DNA	None	6
NCT03674073	Phase 1	Recruiting	Hepatocellular carcinoma	DC	None	24
NCT04105582	Phase 1	Recruiting	Triple negative breast cancer	DC	None	5
NCT04078269	Phase 1	Recruiting	Non small cell lung cancer	DC	None	6
NCT04147078	Phase 1	Recruiting	Solid tumors	DC	None	80

EGFR, Epidermal growth factor receptor; TKI, Tyrosine-kinase inhibitor; DC, Dendritic Cell.

### Adoptive Cell Therapies

Another neoantigen-targeted treatment approach is adoptive cell therapy (ACT). Natural or modified T cells are expanded *ex vivo* and infused into patients to enhance T cell responses and kill tumor cells. Adoptive T cell therapies include the adoptive transfer of TILs, or of T cells genetically engineered to express a T cell receptor (TCR), or a chimeric antigen receptor (CAR), as well as other immune cells like natural killer cells (61).

Personal TILs could recognize neoepitopes derived from somatic mutations were identified in gastrointestinal cancers (62). Several studies have shown that the adoptive transfer of T cells specific against oncogenic mutations could mediate tumor regression in metastatic cholangiocarcinoma (22), colorectal cancer (26), cervical cancer (63), and breast cancer (64). In 2014, Rosenberg et al. administrated neoantigen-reactive CD4+ TILs in a patient with metastatic cholangiocarcinoma (NCT01174121), resulting in complete tumor regression (22). This finding evidenced that CD4+ T cells against neoantigens can be used to mediate epithelial cancer regression. Then a patient with metastatic colorectal cancer was found to have CD8+ T cells in TILs which could specifically target mutant KRAS G12D (NCT01174121) (26). After infusion of the HLA-C*08:02–restricted TILs, all lung metastases’ objective regression was observed. One lesion that progressed nine months later was proven to have the loss of chromosome 6 encoding the HLA-C*08:02 MHC class I molecule (26). Additionally, therapeutic TILs against mutant neoantigens induced immunodominant antitumor T cell responses instead of against human papillomavirus (HPV) antigens, resulting in complete tumor regression in virally associated cervical cancer (63). Successful adoptive therapy was also found in patients with metastatic breast cancer (NCT01174121) (64). After received TILs against four neoantigens (SLC3A2, KIAA0368, CADPS2, and CTSB), the patient achieved durable tumor regression over 22 months (64). All these works supported that the adoptive transfer of neoantigen-based TILs played a vital role in immunotherapies.

**Table 2** showed selected ongoing adoptive T cell therapy studies.

**Table 2:** Clinical trial of neoantigen ACT.

ClinicalTrial.gov identifier	Phases	Enrollment status	Cancer type	Additional interventions	Patient accrual target
NCT03374839	Phase 1/2	Recruiting	Melanoma	Nivolumab, IL-2	11
NCT04072263	Phase 1/2	Recruiting	Ovarian cancer	Interferon alfa 2A, carboplatin, paclitaxel	12
NCT03709706	Phase 2	Recruiting	Non small cell lung cancer	Pembrolizumab	54
NCT02926053	Phase 1	Recruiting	Renal cell carcinoma	Fludarabine, cyclophosphamide, IL-2	6
NCT03967223	Phase 2	Recruiting	Solid tumors	Fludarabine, cyclophosphamide	65
NCT04555551	Phase 1	Recruiting	Multiple myeloma	None	36
NCT03970382	Phase 1	Recruiting	Solid tumors	Nivolumab, IL-2	148
NCT03754764	Phase 1/2	Recruiting	Lymphoblastic leukemia	None	80
NCT04526509	Phase 1	Recruiting	Solid tumors	Fludarabine, cyclophosphamide	38
NCT03720496	Phase 1	Recruiting	Non-Hodgkin lymphoma	None	6
NCT04217473	Phase 1	Recruiting	Melanoma	None	15
NCT02870244	Phase 1	Recruiting	Melanoma	None	18
NCT03497533	Phase 1/2	Recruiting	Non-Hodgkin lymphoma	None	6
NCT04430595	Phase 1/2	Recruiting	Breast cancer	None	100
NCT04571138	Phase 1/2	Recruiting	Leukemia, lymphoma	None	42
NCT04729543	Phase 1/2	Recruiting	Melanoma, head-and-neck carcinoma	None	20
NCT04596033	Phase 1	Recruiting	Solid tumors	Fludarabine, cyclophosphamide, IL-2	24
NCT00881920	Phase 1	Recruiting	Lymphoma, myeloma, leukemia	None	54
NCT03638167	Phase 1	Recruiting	Pediatric central nervous system tumor	None	36
NCT03500991	Phase 1	Recruiting	Pediatric central nervous system tumor	None	48
NCT03326921	Phase 1	Recruiting	Acute Leukemia	Fludarabine	24
NCT03318900	Phase 1	Recruiting	Ovarian carcinoma	Cyclophosphamide, utomilumab, IL-2	18
NCT03412526	Phase 2	Recruiting	Ovarian Cancer	Fludarabine, IL-2	15
NCT03338972	Phase 1	Recruiting	Multiple myeloma	Fludarabine, cyclophosphamide	25
NCT03186118	Phase 1	Recruiting	Acute Leukemia	None	30
NCT03166878	Phase 1/2	Recruiting	Leukemia, Lymphoma	None	80
NCT03330691	Phase 1	Recruiting	Leukemia	None	46
NCT02028455	Phase 1/2	Recruiting	Leukemia	None	80
NCT03823365	Phase 1	Recruiting	Non-Hodgkin lymphomas, chronic lymphocytic leukemia	None	13
NCT01955460	Phase 1	Recruiting	Melanoma	Fludarabine, cyclophosphamide, IL-2	15
NCT03434769	Phase 1	Recruiting	Non-Hodgkin lymphoma	Fludarabine, cyclophosphamide	18

## Challenges

Despite recent advances, many challenges remain for the application of personalized neoantigen-based vaccine or adoptive cell transfer.

A critical issue that needs to be addressed is the expensive and time-consuming manufacturing. Although the cost of genome sequencing has decreased (65), it remains costly to identify neoantigens and process good manufacturing practices. The overall timeline from acquiring the patient’s sample to vaccine administration was about 3 to 5 months (34). Reducing production turnaround time is urgent, especially for patients with metastatic disease. These cell-based experiments also are difficult to standardize and require significant numbers of cells. Thus high-throughput and unbiased computational strategies may need to select neoantigens (4). An additional obstacle could be neoantigens prediction and validation. Despite the current computational neoantigen prediction algorithms and experimental validation approaches (tetramers or multimers, ELISpot) used to prioritize neoantigens, some efforts are still needed to pursue further optimization. Strategies include better predict MHC-peptide binding and develop big datasets and new algorithms (4). In addition, tumor heterogeneity is common and can be caused by several factors, including 1) spontaneous mutations during tumor progression, 2) tumor microenvironments regulation or neoantigen loss (66), and 3) multiple lesions or even an individual tumor originated from different subclones (61). Tumor heterogeneity can reduce the accuracy of antigen clone prediction in heterogeneous tumor masses. Therefore, it is vital to analyze beneficial mutations carefully. In addition, fully personalized immunotherapy targeting multiple clonal neoantigens may also need to overcome the obstacle of tumor heterogeneity (4, 61). Another challenge is to define the reliable immune biomarkers to predict antitumor immunity and even survival benefit. Although immune-related response criteria (irRC) attempt to evaluate immunotherapeutic effects in clinical practice, they may still not fully reflect all the characteristics of clinical responses (67). Furthermore, the T cell responses induced by neoantigens-based therapies may not directly be translated into durable clinical responses. Thus, it is plausible to identify immune response biomarkers in a systematic pattern.

## Conclusions

Emerging evidence reveals that tumor neoantigens play an essential role in antitumor immunity and successful cancer immunotherapies. Both personalized neoantigen-based vaccines and ACT approaches have shown encouraging antitumor results. Decreased production turnaround time, reduced manufacturing cost, better detection of immunogenic neoantigens, improved computational algorithms, and effective treatment biomarkers are expected to add the feasibility, affordability, and momentum of neoantigen targeting therapies. Neoantigen-based therapies have the potential to turn “cold” tumors into “hot” ones. Therefore, it’s warranted to explore the combinatorial approaches with other immunotherapies, including checkpoint blockade therapies or conventional treatments, including chemoradiotherapies, kinase inhibitors, anti-angiogenesis therapies, et al. (68). Thus, it’s plausible to think that neoantigen-based tailored therapies can be widely performed in various cancers soon.

## Author Contributions

YZ: Conceptualization, writing the original draft. JL: Funding acquisition, writing review, and editing. All authors contributed to the article and approved the submitted version.

## Funding

This work was supported by the Sichuan Science and Technology Department Key Research and Development Project (2019YFS0539), the 1.3.5 Project for Disciplines of Excellence, West China Hospital, Sichuan University (ZYJC18022), and the National Clinical Research Center for Geriatrics (West China Hospital, Z2018B12).

## Conflict of Interest

The authors declare that the research was conducted in the absence of any commercial or financial relationships that could be construed as a potential conflict of interest.

## Publisher’s Note

All claims expressed in this article are solely those of the authors and do not necessarily represent those of their affiliated organizations, or those of the publisher, the editors and the reviewers. Any product that may be evaluated in this article, or claim that may be made by its manufacturer, is not guaranteed or endorsed by the publisher.
